# Utilization of antenatal ultrasound scan and implications for caesarean section: a cross-sectional study in rural Eastern China

**DOI:** 10.1186/1472-6963-12-93

**Published:** 2012-04-12

**Authors:** Kun Huang, Fangbiao Tao, Joanna Raven, Liu Liu, Xiaoyan Wu, Shenglan Tang

**Affiliations:** 1 School of Public Health, Anhui Medical University, Hefei city, Anhui Province, People's Republic of China; 2 International Health Group, Liverpool School of Tropical Medicine, Liverpool, UK; 3 Duke Global Health Institute, Duke University, Durham, North Carolina, USA

**Keywords:** Ultrasonography, Prenatal, Caesarean Section, Rural Health, China

## Abstract

**Background:**

Antenatal ultrasound scan is a widely accepted component of antenatal care. Studies have looked at the relationship between ultrasound scanning and caesarean section (CS) in certain groups of women in China. However, there are limited data on the utilization of antenatal ultrasound scanning in the general population, including its association with CS. The purpose of this study is to describe the utilization of antenatal ultrasound screening in rural Eastern China and to explore the association between antenatal ultrasound scan and uptake of CS.

**Methods:**

Based on a cluster randomized sample, a total of 2326 women with childbirth participated in the study. A household survey was conducted to collect socio-economic information, obstetric history and utilization of maternal health services.

**Results:**

Coverage of antenatal care was 96.8% (2251/2326). During antenatal care, 96.1% (2164/2251) women received ultrasound screening and the reported average number was 2.55. 46.8% women received at least 3 ultrasound scans and the maximum number reached 11. The CS rate was found to be 54.8% (1275/2326). After adjusting for socio-demographic and clinical variables, it showed a statistically significant association between antenatal ultrasound scans and uptake of CS by multivariate logistic regression model. High husband education level, high maternal age, having previous adverse pregnant outcome and pregnancy complications during the index pregnancy were also found to be risk factors of choosing a CS.

**Conclusions:**

A high use of antenatal ultrasound scan in rural Eastern China is found and is influenced by socio-demographic and clinical factors. Evidence-based guidelines for antenatal ultrasound scans need to be developed and disseminated to clinicians including physicians, nurses and sonographers. Guidance about the appropriate use of ultrasound scans should also be shared with women in order to discourage unreasonable expectations and demands. It is important to monitor the use of antenatal ultrasound scan as well as the indications for caesarean section in rural China.

## Background

Countries with high coverage of skilled maternal care often face problems related to frequent utilization of instrumental intervention, the commonest two are high rate of Caesarean Section (CS) and overuse of antenatal ultrasound scans [1].

CS is needed to prevent or treat life-threatening maternal or foetal complications in an estimated 5-15% of pregnancies [2]. Globally there are great discrepancies in the availability and use of CS with inability to meet these minimum coverage levels in many low to middle income countries and increasing concern about rising CS rates in other areas. This was recently highlighted in surveys from Asia and Latin America [3-5]. Of 60 medium and high income countries, the majority (62%) had national rates of cesarean section above 15% [6]. For Asia the overall CS rate was estimated to be 27.3% with the highest estimate for China at 46.2% [5]. In China rates are reported to be increasing twice as much in rural areas compared to urban areas [7,8].

Increasing CS rates do not necessarily lead to improved outcomes and may be associated with increased risk of maternal mortality, hysterectomy, haemorrhage, infection, ureteral tract injury, neonatal respiratory morbidity and placenta praevia and uterine rupture in future pregnancies [4,9]. In addition increasing CS rates can be considered a resource drain especially where resources are scarce.

Many factors have influenced this change in practice including increasing consumer awareness and expectation, fear of intrauterine death, brain injury and pelvic floor damage associated with vaginal delivery, beliefs requiring a specific day and time for childbirth and physicians' consideration of financial benefits [10-14]. In many maternal health institutions, especially rural settings, providers' poor skills of natural birth attendance as well as insufficient abilities to identify mother's and fetal's abnormalities in pregnancy and delivery can also cause high use of CS and technological antenatal care [15].

Since being introduced to obstetrics in the 1970s, antenatal ultrasound scan is widely used for confirmation of viability of pregnancy and gestational age, identification of multiple pregnancy, and screening for fetal anomalies [16]. It is very typical for women with normal pregnancies to have multiple ultrasound examinations. For example, in the United States, both low-risk and high-risk pregnant women are reported to be more likely to receive repeated ultrasound examinations today than they were 10 years ago. The overall estimated average number of ultrasounds per pregnancy increased from 1.5 in 1995 to 2.7 in 2005 [17]. The average number of scans per woman varies amongst countries. In the United Kingdom, the average number of scans was reported to be 2.6 per woman [18], whilst in Iran the average number is reported to be as high as 5.9 per woman [19].

The routine utilization of ultrasound has widely occurred in Euro-America and Asia. An imaging workshop organized by the Eunice Kennedy Shriver National Institute of Child Health and Human Development in the United States reached a consensus that all pregnant women should be offered an ultrasound scan for the detection of fetal anomalies and pregnancy complications [20]. There are debates whether benefits of routine ultrasound justify their costs. Two reviews suggest that routine ultrasound screening is unlikely to be more beneficial or cost-effective than targeted screening of women with specific risk factors [21,22]. These views are similar to those of the National Institute of Health, the American College of Obstetricians and Gynecologists [23], and the American Institute of Ultrasound in Medicine [24]. In the United States, estimates per one million women screened have ranged from 200 to 500 million dollars annually [25]. As ultrasound technology improves and utilization of ultrasound increases, health-care systems will undoubtedly face even higher associated costs [26,27]. This is of particular importance in low income settings where scarce resources need to be carefully allocated.

Torloni MR and colleagures [28], on behalf of ISUOG (International Society of Ultrasound in Obstetrics and Gynecology)-WHO fetal growth study group completed a systematic review and meta-analysis on safety of ultrasound in pregnancy. The authors said that ultrasonography in pregnancy was not associated with adverse maternal or perinatal outcome, impaired physical or neurological development and subnormal intellectual performance or mental diseases. Although they concluded that exposure to diagnostic ultrasonography during pregnancy "appeared" to be safe, they also pointed out that the studies this systematic review included were mostly published before 1995, when the acoustic potency of the equipment used was lower than in modern machines. Over the years, there has been a continuous trend of increasing acoustic output. In a new meta-analysis, it was found that non-right handedness among all children enclosed to ultrasound in pregnancy was significantly increased [29,30]. Current evidence suggests that the use of Doppler ultrasound in high-risk pregnancies reduced the risk of perinatal deaths [31], but routine fetal and umbilical Doppler ultrasound examination in low-risk or unselected populations did not result in increased antenatal, obstetric and neonatal interventions, and no overall differences were detected for substantive short term clinical outcomes [32]. Recently, Salvesen KA et al. advocate that Doppler examination of fetal vessels in early pregnancy should not be performed without a clinical indication because Doppler usually generates higher intensity outputs than does B-mode ultrasound and people now still live with uncertainty regarding ultrasound safety [33]. Thus current epidemiological evidence is not synchronous with advancing ultrasound technology and an absence of evidence of harm is not equal to evidence of absence of harm.

Psychological effects of ultrasound imaging on pregnant women are also well documented in the literature, which might be a bridge of linkage between antenatal ultrasound scan and CS decision. An ultrasound examination has the potential to be a fascinating and happy experience for prospective mothers and couples. However, it may also have a disturbing effect and significantly increase mothers' anxiety levels in cases where the test results are true- or false-positive regarding fetal abnormalities resulting in changes in health care behaviors of women such as concern about life style [34] or terminating pregnancy by caesarean sections (CS) [35,36]. Studies have described the difference in CS rate between women who received ultrasound examination and those who did not. They also found that ultrasound scan in identifying estimated fetal weight and nuchal cord problems result in more CS [35,36]. Even in non-macrosomic neonates, the antenatal ultrasonographic diagnosis of suspected macrosomia is associated with a significant increase in CS rates [36].

As ultrasound scans are overused in many countries under the condition of unclear safety and may cause more medical interventions from negative psychological effects, more emphasis should be attached on the relationship between antenatal ultrasound scanning and CS. However, there are few studies with specific objective to examine the attribution of antenatal ultrasound to CS. In China, researchers conducted a few studies in certain groups of women and found increased CS rate in women with reported nuchal cord and macrosomia by ultrasound scan [37,38]. But they only provided limited evidence of the association and there are lack of data with general women to explain the association between antenatal ultrasound and CS. China has the largest agricultural population in the world with relatively limited health resources and a rapidly increasing CS rate in rural areas. Since there is no firm evidence to show that it is at zero risk to expose to antenatal ultrasound scan, we conducted this study aiming to: (1) describe the utilization of antenatal ultrasound scan in rural China; (2) explore the association between antenatal ultrasound scan and CS. We hope it will attract maternal policy-makers' more attention on this issue and provide evidence for their decision-making in rural China.

## Methods

Ethical approval of this study was obtained from Biomedicine Ethical Committee in Anhui Medical University (Approval No. 2007002). Written informed consent was obtained from the participants for publication of this report and any accompanying images.

### Data collection

CHIMACA is an international collaborative project intended to find out the hinders in good maternal care in rural China and accordingly frame community interventions to improve access and quality of maternal health care. To better evaluate the effects of intervention and avoid the "contaminations" among programs as well, within Anhui province in Eastern China, two counties were selected using the following criteria: 1) the local government was interested in the project and willing to participate; 2) Except for New Collaborative Medical Scheme (NCMS) (NCMS was a required condition for the study sites because one intervention was made based on the NCMS system), there were no other maternal health care improvement programs ongoing in the counties at the time of the study; 3) there were adequate numbers of population and townships. Within the two counties, 30 townships were selected: all 18 townships in FC county; and 12 out of 20 townships by geographic characteristics and distance in XC county.

**Figure 1:**
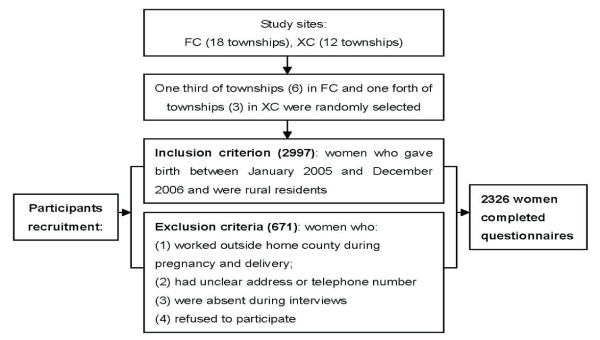
**Flow diagram showing recruitment of research participants**.

A household survey was conducted in December 2006. Six of the 18 townships in FC county and 3 of the 12 townships in XC county were randomly selected. For all villages in these townships, all women who gave birth between January 2005 and December 2006 and were rural residents were identified, traced and recruited as participants. Participant recruitment is shown in Figure 1. Participants were interviewed using a structured questionnaire which included general demographic information, socioeconomic status, general medical and obstetric history, and utilization of maternal health services in the index pregnancy.

### Data analysis

Questionnaire coding and data entry were performed with EpiData 3.0. Statistical analysis was carried out using the SPSS (version 16.0) software packages. Having reviewed the key literature and carefully considered the situation in China, the following socio-demographic and clinical variables were selected from the questionnaire as being the most likely to be associated with the use of CS: maternal age, maternal educational years, husband educational years, family annual income (classified as 1. ≤ 3000 yuan, 2.3001-7499 yuan, 3. ≥ 7500 yuan), ultrasound scans during the index pregnancy, primiparity, previous adverse pregnant outcome (including abortion, stillbirth and fetal death), pregnancy complications (including placenta praevia, polyhydramnios, oligohydramnios uterus myoma, ovarian cyst, hypertension, diabetes, hepatitis, nephritis, acute appendicitis, anaemia, intrahepatic cholestasis of pregnancy, blood type incompatibility), antenatal care (antenatal check-up during the 1st trimester of pregnancy plus 4 times during 2nd and 3 rd trimesters), preterm delivery (gestational age < 37 weeks) and large for date infant (birth weight ≥ 4 kg). Analysis of variance (one-way ANOVA) and *χ*^2 ^tests were adopted to explore differences of socio-demographic and clinical indicators with various antenatal ultrasound scans.

By using univariate logistic regression model, associations between all above-mentioned variables and CS were displayed. Uptake of CS was regarded as dependent variable, antenatal ultrasound scans and other variables were introduced into the model as independent variable one by one. Crude odds rations, OR 95% CI and *p *values were described. Then further multivariate logistic regression models were adopted to explore the association between antenatal ultrasound scans and uptake of CS. Variables with *p *values over 0.1 in univariate logistic regression model were excluded from the multivariate logistic regression analysis. Except for antenatal ultrasound scans, the remaining variables were stratified into two groups: socio-demographic variables and clinical variables. In the original model, ultrasound scan was inserted as the only independent variable. Socio-demographic variables were then added into the first model and clinical variables were added into the second model. In the last model, both socio-demographic and clinical variables were simultaneously introduced into the model as independent variables. Crude OR and adjusted ORs for association between antenatal ultrasound scans and uptake of CS were observed. The multivariate logistic regression anakysis was performed with the method Forward: LR and the statistical significance was set at the alpha = 0.05 level.

## Results

### Utilization of antenatal care and ultrasound scan

A total of 2326 women were interviewed using the structured questionnaire. The loss rate was 22.4% (Figure 1). The coverage of antenatal care was found to be 96.8% (2251/2326). During antenatal care, 96.1% (2164/2251) women received ultrasound scans and the average reported number of scans was 2.55. 46.8% women were screened for 3 or more times with the maximum number being 11 scans.

Socio-demographic and clinical factors were explored in relation to number of ultrasound scans (Table 1). By *χ*^2 ^tests, it showed that in both counties, women with younger age, higher education level of women and husband, higher annual income, primiparous women and women who had more antenatal care, pregnancy complications were more likely to have more frequent ultrasound scans, especially with scans over 3 times. More scans women had during index pregnancy, more possibly they would choose a CS.

**Table 1:** Association between number of ultrasound scans and socio-demographic and clinical variables (n/%)

Variables	Number of ultrasound scans			*P *value
		
	No scans	1-2	≥ 3	
Maternal age (years)				< 0.001

≤ 23 years	36/22.2	53/32.7	73/45.1	

24-31 years	249/23.2	512/47.6	314/29.2	

≥ 32 years	313/28.7	544/50.0	232/21.3	

Maternal education level (years)				< 0.001

≤ 5 education years	95/58.6	58/35.8	9/5.6	

6-8 education years	451/42.0	512/47.6	112/10.4	

≥ 9 education years	317/29.1	573/52.6	199/18.3	

Husband education level (years)				< 0.001

≤ 5 education years	49/30.2	93/57.4	20/12.3	

6-8 education years	233/21.7	675/62.8	167/15.5	

≥ 9 education years	191/17.5	655/60.1	243/22.3	

Family annual income (yuan)				< 0.001

≤ 3000 yuan	117/72.2	34/21.0	11/6.8	

3001-7499 yuan	614/57.1	294/27.3	167/15.5	

≥ 7500 yuan	556/51.1	359/33.0	174/16.0	

Antenatal care (n/%) *				< 0.001

Yes	17/2.0	294/35.4	519/62.5	

No	145/9.7	781/52.2	570/38.1	

Adverse outcomes in previous pregnancy (n/%)				0.001

Yes	11/6.7	54/33.1	98/60.1	

No	151/7.0	1021/47.2	991/45.8	

Pregnancy complication (n/%)				< 0.001

Yes	15/2.9	208/40.8	287/56.3	

No	147/8.1	867/47.7	802/44.2	

Primiparity (n/%)				< 0.001

Yes	74/4.7	681/42.9	834/52.5	

No	88/11.9	394/53.5	255/34.6	

Preterm delivery (n/%)				0.710

Yes	11/7.9	68/48.6	61/43.6	

No	151/6.9	1007/46.1	1028/47.0	

Large for date infant (n/%)				< 0.001

Yes	31/14.0	93/41.9	98/44.1	

No	131/6.2	982/46.7	991/47.1	

Caesarean section (n/%)				< 0.001

Yes	56/4.4	509/39.9	710/55.7	

No	106/10.1	566/53.9	379/36.1	

* Antenatal care refers to antenatal check-up during the 1st trimester of pregnancy plus 4 times during 2nd and 3 rd trimesters, ie, at least 5 antenatal visits during pregnancy

### CS rates and decision making around CS

The CS rate was found to be 54.8% (1275/2326), in which primiparity accounted for 70.4% (898/1274). Using the structured questionnaire, women who had undergone CS were asked to identify who had been the key decision maker with regard to their mode of delivery. The decision to have a caesarean delivery was taken by 50.6% of women (645/1275), 43.8% of doctors (559/1275) and 5.6% (71/1275) of other persons, including family members, relatives and friends. Women reported fear of pain and beliefs that caesarean section was safer for both mother and baby as the most common reasons for choosing CS.

### Association between ultrasound scan during pregnancy and uptake of CS

By using univariate logistic regression model, it was found that high maternal and husband education level, high family annual income, having antenatal care, more frequent ultrasound scans during frequency, having pregnancy complications, previous adverse prenant outcomes and primiparity were related with more likelihood of CS. When ultrasound scan was inserted as the only independent variable, there was a strong statistical significance between antenatal ultrasound scans and CS (OR: 1.342, 95%CI: 1.265-1.423). Table 2.

**Table 2:** Univariate logistic regression analysis of variables associated with CS

Variables	Crude OR	OR 95% CI	*P *value
Maternal education level (years)*	1.060	1.032-1.088	< 0.001

Husband education level (years)*	1.069	1.031-1.109	< 0.001

Maternal age (24-31 y as control)			

≤ 23 y	0.830	0.680-1.103	0.067

≥ 32 y	1.058	0.868-1.290	0.576

Family annual income (≤ 3000 yuan as control)			

3001-7499 yuan	1.316	1.091-1.586	0.004

≥ 7500 yuan	1.388	1.092-1.763	0.007

Antenatal care^#^			

No	-	-	-

Yes	1.356	1.142-1.611	0.001

Antenatal ultrasound scans (frequencies)*^&^	1.342	1.265-1.423	< 0.001

Pregnancy complications			

No	-	-	-

Yes	1.601	1.308-1.961	< 0.001

Previous adverse pregnant outcomes			

No	-	-	-

Yes	1.726	1.232-2.417	0.001

Primiparity			

No	-	-	-

Yes	1.241	1.042-1.479	0.016

Preterm deliveries			

No	-	-	-

Yes	0.865	0.615-1.218	0.407

Large for date infants			

No	-	-	-

Yes	0.875	0.663-1.154	0.343

* continuous variables

^# ^Antenatal care refers to antenatal check-up during the 1st trimester of pregnancy plus 4 times during 2nd and 3 rd trimesters, ie, at least 5 antenatal visits during pregnancy

^&^Antenatal ultrasound scans means the frequencies that women had ultrasound scans during the index pregnancy

The association between antenatal ultrasound scans and CS was further explored by adopting multivariate logistic regression analysis. Preterm deliveries and large for date infants were excluded with the *p *value of 0.407 and 0.343, respectively. Then the indepentent variables were classified into two groups: socio-demographic variables (including maternal and husband education level, maternal age and family annual income) and clinical variables (including antenatal care, pregnancy complications, previous adverse pregnant outcomes and primiparity). When adjusting for socio-demographic variables, the odds ratio of antenatal ultrasound scans was 1.346 (95%CI: 1.267-1.429). When adjusting for clinical variables, the odds ratio was 1.323 (95%CI: 1.247-1.404). When both socio-demographic and clinical variables were included in the model, the odds ratio of antenatal ultrasound scans was 1.319 (95%CI: 1.241-1.401). (Table 3).

**Table 3:** Multivariate logistic regression analysis of variables associated with CS

Variables	Crude OR	Adjusted OR _1_	Adjusted OR _2_	Adjusted OR _3_
Antenatal ultrasound scans (frequencies)*^&^	1.342 (1.265-1.423)	1.346 (1.267-1.429)	1.323 (1.247-1.404)	1.319 (1.241-1.401)

Maternal education level (years)*	-	-	-	

Husband education level (years)*	-	1.058(1.018-1.099)	-	1.059(1.019-1.101)

Maternal age (24-31 y as control)				

≤ 23 y	-	0.790(0.643-0.971)	-	0.766(0.641-0.943)

≥ 32 y	-	1.294(1.049-1.595)	-	1.441(1.129-1.840)

Family annual income (≤ 3000 yuan as control)				

3001-7499 yuan.	-	1.225(1.008-1.487)	-	

≥ 7500 yuan	-	1.294(1.008-1.661)	-	

Antenatal care^# ^(No as control)				

Yes	-	-		

Pregnancy complications (No as control)				

Yes	-	-	1.449(1.177-1.783)	1.423(1.154-1.754)

Previous adverse pregnant outcomes (No as control)				

Yes	-	-	1.584(1.107-2.211)	1.584(1.117-2.246)

Primiparity (No as control)				

Yes	-	-		1.362(1.079-1.718)

Crude OR: only antenatal ultrasound scans as the independent variable

Adjusted OR_1_: adjusted for socio-demographic variables, including maternal and husband education level, maternal age and family annual income

Adjusted OR_2_: adjusted for clinical variables, including antenatal care, pregnancy complications, previous adverse pregnant outcomes and primiparity

Adjusted OR_3_: adjusted for both socio-demographic and clinical variables, including maternal and husband education level, maternal age, family annual income, antenatal care, pregnancy complications, previous adverse pregnant outcomes and primiparity

* continuous variables

^# ^Antenatal care refers to antenatal check-up during the 1st trimester of pregnancy plus 4 times during 2nd and 3 rd trimesters, ie, at least 5 antenatal visits during pregnancy

^&^Antenatal ultrasound scans means the frequencies that women had ultrasound scans during the index pregnancy

When both socio-demographic and clinical variables were introduced to the logistic regression model, high husband education level, older maternal age, women who had adverse outcomes in previous pregnancies, had pregnancy complications in the index pregnancy and primiparity were all independently associated with CS. (Table 3).

## Discussion

This paper reports a study in rural Eastern China where the use of antenatal ultrasound scans is frequent with 96.1% women reporting having received at least one scan during pregnancy. The average number of ultrasound scans per woman is shown to be 2.55 with a maximum number reaching 11. These findings are consistent with studies in the United States and United Kingdom [17,18]. In another study in China, it is found that women who give birth at home have an average of 2.3 ultrasound scans, whilst women delivering in hospital tend to have more antenatal visits and more ultrasound scans [39]. Higher average numbers of ultrasound scans in pregnancy are seen in Iran and Syria [19,40].

### Possible causes of high rate of antenatal ultrasound scans

Reasons for high antenatal ultrasound use are complex. Without solid evidence on the effects of antenatal ultrasound scans, it makes informed decisions difficult for health care providers who prescribe ultrasound scan. Health care providers' ability to identify and interpret antenatal complications can affect the utilization of ultrasound scans. A qualitative study carried out by our research team indicates that township doctors have poor clinical skills in maternal health [15]. It is possible that doctors, when they do not have sufficient skills to assess the condition of the mother and fetus and communicate these findings, they may rely on interventions such as ultrasound scanning to reassure women and their families. This over-reliance on technology can de-skill clinicians. On the other hand, as salaries for staff in township hospitals are not fully funded by the government and hospitals must generate income themselves, ultrasound scan can be seen as a way of generating income for the health care facility as well as for individual clinicians [15].

Internationally, women rate ultrasound during pregnancy as one of the most important aspects of their antenatal care [41]. Meeting and connecting with the baby, need for reassurance, finding out the malformations are the main reasons why women like to have a scan [42]. A systematic review reports that the attractiveness of ultrasound outweighs other concerns expressed by women, such as safety, over-medicalization and excessive use [41]. Researchers find that many women lack information about the purposes of ultrasound scan and the technical limitations of the procedure. Majority of women believe that ultrasound scanning can detect all types of malformations in the fetus [19]. In China, women also place a high value on ultrasound and take it for granted that a "normal" ultrasound means a "normal" delivery [39]. Additionally, the lack of knowledge of what constitutes quality services is likely to be a contributory factor in the common perception by women that instrumental examination and surgical intervention at birth is indicative of quality [43]. The availability and widespread use of ultrasound scanning as well as CS could be "a marker for a type of patient who prefers medical intervention" [44]. This has also been found in Viet Nam, where the belief that ultrasound scans are considered a sign of quality of care contributed to the overuse [45].

It is found in this study that maternal use of antenatal ultrasound scans are influenced by their socio-demographic characteristics and clinical factors. Younger and primiparous women are more likely to have more frequent antenatal ultrasound scans. It is possibly because that young women in their first pregnancy experience stronger feelings and have more fear of childbirth than women with previous deliveries [46], bringing greater interest to see the baby and assure the baby's health by ultrasound scans. Women and husband with high educational level receive more ultrasound scans because they may be more capable of identifying pregnancy complications and thus request ultrasound scans to be done for reassurance. Frequent antenatal visits also create more opportunities whereby women may request or be prescribed ultrasound scans. A further qualitative design is required to get women's insights into antenatal ultrasound scans in rural China,

### Association between frequent antenatal ultrasound scans and high use of CS

In this study we found that there was a significant association between antenatal ultrasound scan and uptake of CS. We suggest that this is due to two main reasons. Firstly, having an ultrasound scan may create anxiety for the mother. Although the scan can result in feelings of satisfaction and comfort when findings are normal, it also creates extra tensions because of the immediate knowledge gained and the possibility of worrying news. Features of ultrasound that provide people with constant visual confirmation of pregnancy may augment the potential for feelings of anxiety, shock, and disappointment when the scan shows a problem or fails to obtain the necessary information [41,47]. In this study, Concern of the safety for both mother and baby was an important reason for choosing CS, thus women who had uncertain or negative information from scans would prefer CS.

Secondly, high-risk conditions identified by the scan not only add stress to the pregnant women and their partners but also to physicians. Additional scans may be prescribed when there are abnormal findings e.g. nuchal cord or fetal weight is estimated to be high. Dang et al. [37] found a significant increase in CS and operative delivery between women with nuchal cord (one loop) and women without nuchal cord. Other researches described similar results [35], especially in nulliparous women [48]. In many cases nuchal cord does not appear to cause harm. However there are reports that it is associated with an increased risk of fetal distress, higher incidences of low Apgar scores at 1 and 5 minutes, emergency caesarean section, need for assisted ventilation and admission to the neonatal intensive care unit [48,49]. Obstetricians may prefer to suggest an operation rather risk labor in order to avoid medical disputes as well as shirk their own responsibilities. Accuracy of fetal weight estimated by ultrasound scan is controversial [50]. Overestimation of fetal weight by ultrasound may influence the likelihood of CS [51]. Even in non-macrosomic neonates, the antenatal ultrasonographic diagnosis of suspected macrosomia was associated with a significant increase in CS rates [36,38]. It is possible that harm from inappropriate interventions following false-positive diagnoses could cause unnecessary anxiety, outweigh the benefits from appropriate interventions following true-positive diagnoses and lead to over-diagnosis or iatrogenic over-treatment in a condition in which no firm evidence exists.

Ultrasound scan may also indirectly related to CS through identifying more pregnancy complications. Diagnostic ultrasound examination may be employed to help find clinical complications and fetal malformations or inappropriate growth. Although we cannot define the identified disease in each woman, and cannot either tell whether they are identified by ultrasound scans, some diseases might just be clinic indicators for CS or increase the risk of difficult vaginal delivery.

### Other factors related to high CS rate in rural China

Clearly, antenatal ultrasound scans are only one of several associated factors of the increasing and excessive application of CS in rural China. The dramatically uprising CS rates in rural areas happen with the increasing rate of hospital delivery in recent decades. The rate of hospital delivery in both selected counties was 99.2 (2308/2326), almost 100% (not shown in results). Sufang et al. [44] attributed the increase in rates of CS in China to an increase in births within institutions. In addition, higher husband education level, older maternal age, pregnancy complications and previous adverse pregnant outcomes were all related with high use of CS. Pregnancy complications may cause some clinic indicators behind the operation. It also illustrates the high thoughts of maternal and infants' safety if there are previous adverse outcome, resulting in the selection of CS to terminate pregnancy.

Many other reasons, both on user and provider sides, are attributed for the increasing CS rates. Fear of pain and beliefs that CS was safer to both mothers and infants are the two main reasons reported by women choosing a CS. This is in agreement with other international studies, which have found that fear of childbirth is a key factor resulting in women's requests for cesarean section [18,52,53]. Especially nulliparous women had higher scores for fear of childbirth than parous women [46]. Improvement of maternal social status and economic power in China also signify their more involvements in decision-making of delivery mode. Many caesarean sections are performed on maternal request with insufficient information of the risks and health consequences of alternative modes of delivery. A study in Brazil find that vaginal birth is considered a risky and negative experience, whereas cesarean section is regarded to represent the best quality of care [43]. Fear of vaginal delivery is not simply because of poor information on how to prepare for a vaginal birth but relates to women's perceptions that the quality and safety of labor care is poor [43,54].

In guidelines developed by the Royal College of Obstetricians and Gynaecologists (RCOG)and National Collaborating Centre for Women's and Children's Health (NCCWCH), malpresentation, cephalopelvic disproportion and fetal distress are listed as three main clinical indications for CS, nor previous caesarean deliveries. Operations performed from previous delivery by CS, however, are much frequent in developing countries [55,56]. Weak execution of evidence-based maternity care combined with maternal requests contribute to final uptake of CS. Furthermore, provider payment mechanisms and revenue-related bonus payments directly link to the numbers of procedures carried out, and this could be creating a supply-induced demand for the use of more expensive procedures, such as CS [57]. The qualitative study completed by our research team reveals that financial incentives for more hospital profits as well as poor skills of vaginal delivery attendance are main reasons of health providers to prescribe a CS [54]. Defensive medical care (doctor's induced suggestions to avoid disputes from difficult vaginal delivery), beliefs that CS can improve postnatal quality of life are also the common providers' perceptions on CS prescription [54].

China has been experiencing huge social transformation, and culture-dependent factors related to high use of CS must be taken into consideration. For example, maternal request for an auspicious birth date is much common in rural China. And there is irrational social climate of upward comparison in rural areas as well, which regarded CS as the symbol of high economic level and more family's concern on puerpera [54].

### Strengths and limitations

In this paper, the CS rate and utilization of antenatal ultrasound scan are described by clustered randomized sampling, which can produce creditable findings in rural areas of Eastern China. The current study also regards the relationship between antenatal ultrasound scan and CS as the specific objective and provides original population-based evidence of the association between high use of antenatal ultrasound scans and increasing uptake of CS.

There are also several limitations of our paper. Firstly, this study is based on a cross-sectional design. Such a retrospective survey can not allow to conclude definite causality between antenatal ultrasound scan and CS. So what we find are just associations, not explicit causal relations. Secondly, the analyses of CS should be subdivided by whether the delivery was planned before the labour (elective) or not (emergency), and by whether it was performed before the onset of labour (antepartum) or during labour (intrapartum). In our survey, we have to rely on the women's recall and it is difficult to get exact answers because of some women's low educational level, poor medical information and some provider's induction even there were no obvious CS indications. In the third, this study just focuses on women's utilization of antenatal ultrasound scan. Health providers' characteristics, such as physician's demographic data, institution's information, are not systematically collected. Actually, both demand and supply side arguments have been put forward on the use of antenatal ultrasound and CS. Further studies are needed to analyze the integrated effects of both the two sides on maternal technology use. Especially a qualitative research is expected to explore physicians' and women's knowledge of antenatal ultrasound scan, motivations to prescribe/have scans, responses/emotions of assessed fetal development outcomes and the impacts on suggestion/choice of delivery mode.

## Conclusion

In conclusion, a high utilization of antenatal ultrasound scans was observed in this study. Maternal age, maternal and husband education level, family income, parity, antenatal care and pregnancy complications were found to be associated with high ultrasound use. Antenatal ultrasound scan significantly and independently associated with uptake of caesarean section. Evidence-based guidelines for antenatal ultrasound scans need to be developed and disseminated to clinicians including physicians, nurses and sonographers. Guidance about the appropriate use of ultrasound scans should also be shared with women in order to discourage unreasonable expectations and demands. It is important to monitor the use of antenatal ultrasound scan as well as the indications for caesarean section in rural China.

## Competing interests

The authors declare that they have no competing interests.

## Authors' contributions

FT, ST and KH participated in the design of the study. KH, LL and XW were involved in data collection. KH and JR performed statistical analysis and drafted the first manuscript. All authors read, commented on and approved the final manuscript.

## Pre-publication history

The pre-publication history for this paper can be accessed here:

http://www.biomedcentral.com/1472-6963/12/93/prepub
